# Interferon-Lambda 1 Inhibits *Staphylococcus aureus* Colonization in Human Primary Keratinocytes

**DOI:** 10.3389/fphar.2021.652302

**Published:** 2021-03-22

**Authors:** Xia Wu, Yan Zhao, Ying Gu, Kun Li, Xiaojie Wang, Jianzhong Zhang

**Affiliations:** ^1^ Department of Dermatology, Peking University People’s Hospital, Beijing, China; ^2^ Department of Dermatology, Beijing Children’s Hospital, Capital Medical University, National Center for Children’s Health, Beijing, China

**Keywords:** interferon-lambda 1, *Staphylococcus aureus*, atopic dermatitis, keratinocyte, reactive oxygen species, signal transducerand activator of transcription 1

## Abstract

Atopic dermatitis (AD) is a common inflammatory skin disease. *Staphylococcus aureus* (*S. aureus*) colonization in skin lesions occurs in approximately 70% of AD patients. It has been found that IFN-λ1 can inhibit the colonization of *S. aureus* in normal human nasal mucosa. IFN-λ1 can increase IL-28RA in infected human keratinocytes. In this study, we found that IFN-λ1 can increase mRNA expression of FLG and antimicrobial peptides (AMPs) and inhibit TSLP mRNA expression in infected human keratinocytes. IFN-λ1 can increase intracellular ROS level, decrease STAT1 phosphorylation, and inhibit the colonization of *S. aureus* in human primary keratinocytes. These effects were attenuated by knocking-down IL-28R and NADPH oxidase inhibitor, suggesting that this function was mediated by JAK-STAT1 signaling pathway. These results suggest that IFN-λ1 might have an inhibitory effect on *S. aureus* colonization in AD lesions. Our findings might have potential value in the treatment for AD.

## Introduction

Atopic dermatitis is a common inflammatory skin disorder. The prevalence of AD is approximately 30% in children and 10% in adults (Bin et al., 2014; Langan et al., 2020). AD is characterized by eczematous lesions with severe itching and often associated with allergic rhino-conjunctivitis, food allergy and asthma (Alduraywish et al., 2016; Matsumoto et al., 2020). Skin barrier defect, immune dysregulation, environmental factors and microbial dysbiosis contribute to the pathogenesis of AD (Orfali et al., 2019; Ahn et al., 2020). AD is a type 2 inflammatory disease, with increased Th2 cytokine levels, such as interleukin (IL)-4, IL-5, and IL-13 (Eyerich and Novak, 2013). In the acute phase, Th2 and Th22 responses are amplified. In the chronic phase, however, Th1 and Th17 are also activated (Weidinger and Novak, 2016). Increased *Staphylococcus* *aureus* skin colonization is another hallmark of AD, which is often associated with disease severity and exacerbation (Byrd et al., 2017; Tay et al., 2020). Specific immunoglobulin E against *S. aureus* enterotoxin (SE-IgE) has been found in AD patients (Motala et al., 1986). *S. aureus* was found in approximately 70% AD lesions and 39% non-lesional skin (Totte et al., 2016). It has been reported that *S. aureus* can impair barrier function, inhibit antimicrobial peptides (AMP) production, and promote viral loads of HSV-1 and Th2 response (Bin et al., 2012; Geoghegan et al., 2018). Impairment of bacterial clearance might exacerbate the inflammation.

Interferon- lambda (IFN-λ) is a novel type III IFNs, including IFN-λ1 (also known as interleukin (IL)-29), IFN-λ2 (IL-28A), IFN-λ3 (IL-28B) and IFN-λ4 in humans (Broggi et al., 2020). Both type III and type I IFNs (α/β) have antiviral function. Moreover, the type III IFNs are members of the IL-10 related cytokine family (Kotenko et al., 2003; Lazear et al., 2015). IFN-λ has a different conformation from type I IFNs (α/β) and interacts with a heterodimeric receptor (IFN-λR) composed of a unique ligand-binding chain IL-28 receptor A (IL-28RA) and an accessory chain, IL-10 receptor B (IL-10RB), which is shared with the IL-10 receptor and other IL-10-related cytokine receptors (Kotenko et al., 2003). After binding to its receptor, IFN-λ1 activated the Janus kinase-signal transducer and activator of transcription (JAK-STAT) signaling pathway (Sheppard et al., 2003; Zitzmann et al., 2006; Maher et al., 2008). The predominant expression of IFN-λR on respiratory and gastrointestinal epithelial cells (Sommereyns et al., 2008; Banos-Lara Mdel et al., 2015), keratinocytes (KCs) (Witte et al., 2009) and hepatocytes (Marukian et al., 2011) allows strong antiviral effects at local barrier sites without activating a systemic proinflammatory immune response.

IFN-λs have vital functions in defensing against colonization of gram-positive bacteria on epithelial tissues and nasal tissues (Bierne et al., 2012; Lan et al., 2019). Compared with IL-28R^-/-^ mutant mice, the *S. aureus* colonization of wild-type mice had increased after nasal infection with influenza virus, indicating that IL-28R was involved in the colonization of *S. aureus* (Planet et al., 2016). IFN-λ1 was found in the nasal tissue of patients associated with chronic rhinosinusitis with nasal polyps after *S. aureus* infection. However, the antibacterial role was only found in healthy tissue (Lan et al., 2019). The anti-bacterial effect of IFN-λ has been studied in nasal mucosa tissue of IL-28R^-/-^ mouse model and in human nasal tissue. Little is known about the role of IFN-λ in skin *S. aureus* infection. We used human keratinocyte-*S. aureus* infection model to study the role of IFN-λ1 in the immune response to *S. aureus* skin infection. We evaluated the number of *S. aureus* colony forming units (CFU) and ROS expression in keratinocytes in the presence of IFN-λ1, attempting to elucidate the possible mechanism of anti-bacterial effects of IFN-λ1.

## Materials and Methods

### Keratinocyte Culture

Human primary keratinocytes were cultured in keratinocyte culture medium (KCM) (CELLnTEC, CnT-07, Swiss) containing 100 U/ml (penicillin)-100 µg⁄ml (streptomycin) (Invitrogen, 15070063, United States) and 10 µg⁄ml (gentamicin)-250 ng⁄ml (amphotericin B) (Invitrogen, R01510, United States) in a humidified atmosphere of 5% CO_2_ and 37°C. When keratinocytes reached 80% confluency, the culture media were changed to antibiotic-free medium for subsequent experiments.

Keratinocytes were fixed with 4% paraformaldehyde for 15 min at room temperature, then permeabilized with 0.1% Triton X-100. Anti-Cytokertin 5 antibody (1:100, Abcam, ab52635, United Kingdom) and anti-Cytokertin 10 antibody (1:150, Abcam, ab76318, United Kingdom) were applied to keratinocytes and incubated for 1 h. After 3 times washing, goat anti-rabbit IgG (H + L)-CoraLite594 (red) (1:500, Proteintech, SA00013-4, United States) was applied. Images were captured on fluorescence microscope (Leica, DMi8, Germany). DAPI (blue) was used for nuclear counterstain (Supplementary Figure S1).

### Cell Proliferation Assay

The effect of IFN-λ1 to keratinocyte proliferation was measured by Cell Counting kit (CCK-8) assay. Keratinocytes were seeded in a 96-well plate (about 3 × 10^3^ cells per well). After 24 h incubation, IFN-λ1 at the concentrations of 1 ng/ml, 10 ng/ml and 100 ng/ml (Pereprotech, AF-300-02L, United States) were added to the culture media. 10 μl of TransDetect® Cell Counting Kit (CCK) (Transgen, FC101-01, China) was added to each well at the time of 0, 24, 48, and 72 h, respectively. After 1 h incubation, the absorbance at 450 nm was measured by the Multiskan™ FC Microplate Photometer (Thermo Scientific, 51119180, Belgium). Cell colony formation assay was also performed to verify the effect of IFN-λ1 on keratinocyte proliferation. Keratinocytes were planted to 6-well plate at 2,000 cells each well and cultured with various concentration of IFN-λ1 for 15 days. Phosphate buffered saline containing 5% trehalose (5% trehalose-PBS) (MultiSciences, 79-PD0021, China) was used as negative control. After 15 days culture, the cells were fixed with formaldehyde, stained with 0.1% crystal violet, washed with 33% glacial acetic acid and measured with a Multiskan FC microplate photometer (Thermo Scientific, 51119180, Belgium).

### Knockdown of Protein Expression by siRNA Transfection

Three human IL-28RA siRNAs and negative control siRNA were purchased from GenePharma (Figure. S4A). According to the manufacturer’s protocol, negative control siRNA (siNC), IL28RA-1 siRNA (siIL28RA-1), IL28RA-2 siRNA (siIL28RA-2), and IL28RA-3 siRNA (siIL28RA-3) were transfected into keratinocytes using Lipofectamine 3000 Transfection Reagent (Invitrogen, L3000015, United States). After 72 h culture, the cells were harvested for subsequent experiments. Quantitative real-time PCR (qPCR) was used to detect the mRNA level of IL-28RA and western blotting was used to detect the protein level of IL-28RA.

### Preparation of *Staphylococcus aureus*

*Staphylococcus aureus* strain was donated by department of laboratory, Peking University People's Hospital, which was isolated from AD eczematous skin lesions. *S. aureus* were grown in 10% sodium chloride tryptone soy broth (TSB) (Solarbio, LA3750, China) medium for 16–18 h at 37°C under agitation (120 rpm) and collected by centrifugation at 2000 rpm for 10 min at room temperature. After washing three times, *S. aureus* were resuspended in PBS (Gibco, 10010049, United States). The concentration of bacteria was measured by the Multiskan™ FC Microplate Photometer at 600 nm wavelength and diluted properly before infection experiment. The bacteria samples were planted onto the mannitol salt agar (MSA) plate (Solarbio, LA 1980, China) for 24 h at 37°C (Supplementary Figure S2A). The 16s rRNA, femA, and mecA were measured by multiplex PCR with the EmeraldAmp® PCR Master Mix (Supplementary Figure S2B). The primer sequences used in the multiplex PCRs were described in Supplementary Table S1. The sequencing of PCR products was performed by Sangon Biotech (Shanghai, China), and the resulting sequences were compared with *S. aureus* strains in Genebank by using the BLAST online program on the NCBI website (Supplementary Figure S3).

### Keratinocyte Infection Model

Human primary keratinocytes were planted into the 6-well plate and incubated with IFN-λ1 (1 ng/ml, 10 ng/ml and 100 ng/ml) or 5% trehalose-PBS as control for 24 h. *S. aureus* were added to keratinocyte culture at the ratio of 30:1 (bacteria: keratinocytes) and incubated for 3 h (infection model). Keratinocytes without *S. aureus* infection were also incubated in the presence of IFN-λ (non-infection model). After incubation, the infected keratinocytes and non-infected keratinocytes were used for qPCR and western blotting.

In order to study the role of IFN-λ1 in elimination of *S. aureus*, keratinocytes were treated with siIL28RA-1 for 48 h or 10 μM NADPH oxidase inhibitor diphenyleneiodonium chloride (DPI) (APO × BIO, B6326, United States) for 1 h. IFN-λ1 10 ng/ml were then added to culture media and incubated for 24 h before *S. aureus* infection. The keratinocyte lysates were collected for detection of STAT1 phosphorylation. The intracellular ROS expression was measured by flow cytometry and fluorescence microscopy. The colony forming unit (CFU) was also evaluated.

### Quantitative Real-Time PCR

Total RNA was extracted from keratinocytes by using the total RNA isolation kit (Beibei Biotechnology, 082001, China) and was reverse-transcribed by using the TransScript^®^ All-in-one first-strand cDNA synthesis superMix for qPCR kit (Transgen, AT341, China). The mRNA expression of IFN-λ1, IL-28RA, IL-10RB, involucrin (IVL), filaggrin (FLG), thymic stromal lymphopoietin (TSLP), human antimicrobial β-defensin (hBD) 1, hBD2, hBD3, S100 calcium binding protein A7 (S100A7), S100A8 and S100A9 was measured by qPCR, using SYBR^®^ Premix Ex Taq™ II (Takara, RR820A, Japan) on the LightCycler 480 Instrument II (Roche Applied Science, Germany). All primers were listed in Supplementary Table S2. The housekeeping genes (GAPDH and β-actin) were used to normalize transcription and amplification variations among the samples. All primers were purchased from Sangon Biotech (Shanghai, China). Relative expression ratio was calculated using the comparative threshold cycle (Ct) and 2^−ΔΔCt^ method. All experiments were performed in triplicate.

### Western Blotting

The treated keratinocytes were lyzed with RAPI buffer (New Cell & Molecular Biotech, WB3100, China) containing proteinase and phosphatase inhibitor cocktail (Beyotime Biotechnology, P1045, China). Protein of each sample was separated by 10% SDS-PAGE (Biotides Biotech, WB2102, China) and transferred to polyvinylidene fluoride membranes (EMD Millipore, ISEQ00010, United States). The primary antibodies for western blotting included anti-STAT1 (1:1,000, CST, 14995, United States), anti-phospho-STAT1 (Tyr701) (1:1,000, CST, 9167, United States) and anti-IL-28R antibody (1:750, Abcam, ab224395, United Kingdom). The blots were then incubated with anti-rabbit IgG, HRP-linked antibody (1:3,000, CST, 7074, United States). The specific bands were observed by Tanon Imaging System (Tanon, China) with Immobilon Western Chemiluminescent HRP Substrate (Millipore, WBKLS0500, United States). The band intensity was calculated by ImageJ software (National Institutes of Health, Bethesda, MD) compared with GAPDH (1:10,000, Proteintech, 60004-1-lg, United States). The secondary antibody for GAPDH was Goat Anti-Mouse IgG H&L (HRP) (1:9,000, Abcam, ab6789, United Kingdom).

### Cell Apoptosis Assay

Cell apoptosis was measured by TransDetect^®^ Annexin V-FITC/PI Cell Apoptosis Detection Kit (Transgen, FA101, China). After treatment with various concentrations of IFN-λ1 for 24 h and infected by *S. aureus*, keratinocytes (2 × 10^5^ cells) were harvested, resuspended in 100 μl one x Annexin V binding buffer and incubated with 5 μl Annexin V-FITC and 5 μl propidium iodide (PI) for 15 min in the dark at room temperature. The samples were detected by a Beckman Coulter FC500 cytometer immediately, and the FlowJo software version 10.4 (BD, United States) was used for data analysis.

### Measurement of Intracellular ROS Levels

Intracellular ROS levels were measured by Reactive Oxygen Species Assay Kit (Beyotime Biotechnology, S0033S, China). Intracellular ROS is able to oxidize 2′,7′-dichlorofluorescein-diacetate (DCFH-DA) to fluorescent dichlorofluorescein (DCF) to quantify the level of ROS. The keratinocytes were planted to 6-well plates and incubated with DCFH-DA at 37°C for 20 min. The cells were washed three times with Dulbecco’s phosphate buffered saline (DPBS) (Gibco, 14190144, United States). The fluorescence signal intensity and the percentages of positive probe were measured by a Beckman Coulter FC500 cytometer (Beckman, United States) and observed by fluorescence microscope (Leica, DMi8, Germany).

### Colony Formation Assay

For colony formation assay, the infected keratinocytes were harvested and washed three times with DPBS. Each sample was added with 1 ml of cold sterile water and placed on ice for 30 min allowing intracellular *S. aureus* to release. Diluent cell lysate was inoculated on MSA plate at 37°C for 24 h. The number of CFU was counted by counting the number of bacterial communities. The experiment was repeated at least three times.

### Statistical Analyses

All data were analyzed by SPSS Statistics 26.0 software (IBM, United States) and GraphPad Prism software (GraphPad Software, CA). Copmarison to statistical analysis was conducted using one-way ANOVA or LSD test and *p* < 0.05 was considered statistically significant.

## Results

### IFN-λ1 can Increase IL-28RA Expression in Keratinocytes Upon *Staphylococcus aureus*-Infection

**FIGURE 1:**
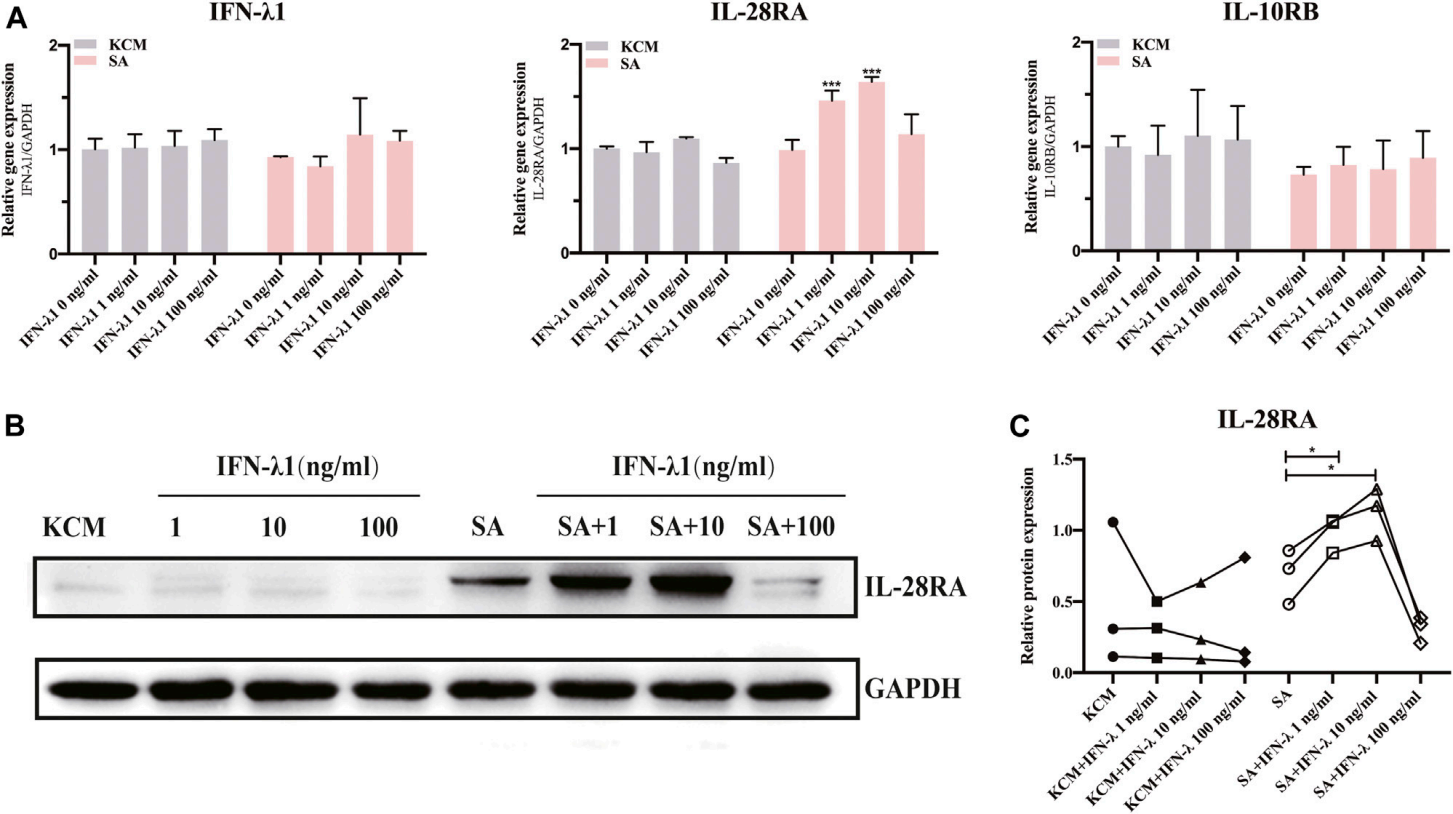
Effects of IFN-λ1 on IL-28RA expression in *S. aureus* infected human keratinocytes. **(A)** Effects of IFN-λ1 on mRNA expression of endogenous IFN-λ1, IL-28RA and IL-10RB. **(B,C)** Effects of IFN-λ1 on IL-28RA production. Human keratinocytes were treated by various concentrations of IFN-λ1 (1 ng/ml, 10 ng/ml and 100 ng/ml). KCM, keratinocyte culture medium; SA, *S. aureus*. Data are means ± SD. (*n* = 3, *p < 0.05, **p < 0.01, ***p < 0.001 vs. SA).

To illustrate whether IFN-λ1 treatment could stimulate endogenous IFN-λ1 secretion, we studied the expression of IFN-λ1 and IL-28R (IL-28RA and IL-10RB) by qPCR and IL-28RA by western blotting. The keratinocytes were pretreated with IFN-λ1 for 24 h, followed by *S. aureus* infection. Low concentration of IFN-λ1 (1 ng/ml and 10 ng/ml) treatment significantly enhanced IL-28RA mRNA and protein expression in infected keratinocytes (*p* < 0.05), while high concentration of IFN-λ1 (100 ng/ml) showed no effect (Figures 1A,B). The endogenous IFN-λ1 and the accessory chain (IL-10RB) mRNA expression remained unchanged (Figure 1A).

### IFN-λ1 Induces FLG Gene Expression and Inhibits TSLP in Human Keratinocytes During *Staphylococcus aureus* Infection

**FIGURE 2:**
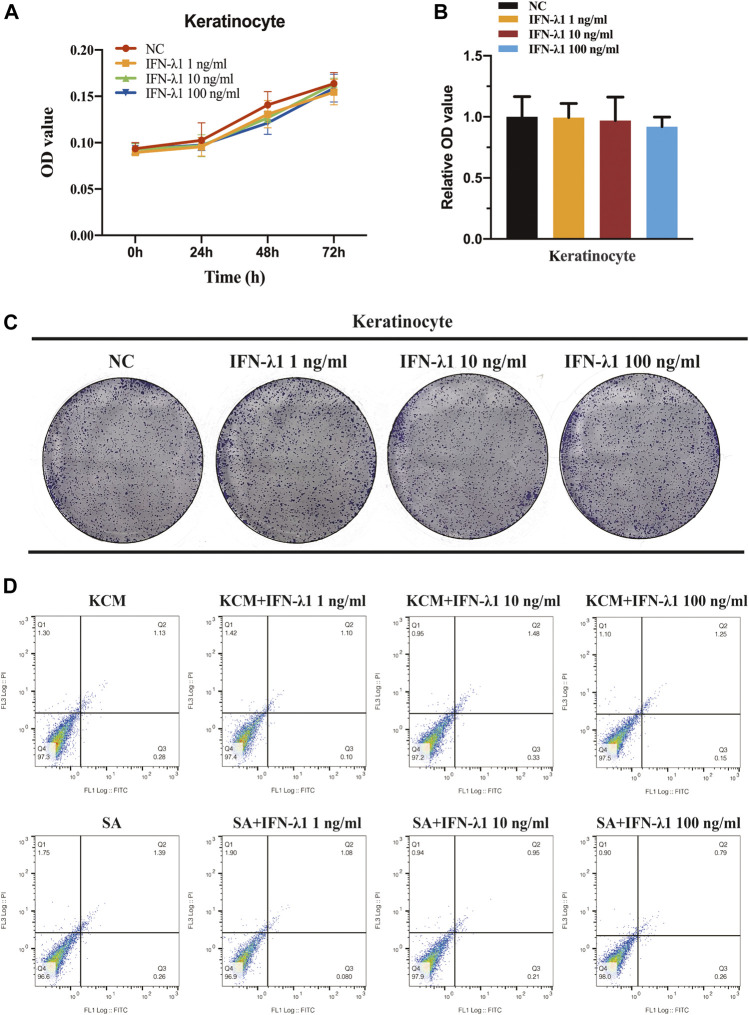
No effect of IFN-λ1 was found on keratinocyte proliferation and apoptosis. **(A–C)** Result of keratinocyte proliferation assays: **(A)** CCK-8 assay; **(B,C)** Colony-formation assay; **(D)** Results of keratinocyte apoptosis assay. Human keratinocytes were treated by various concentrations of IFN-λ1 (1 ng/ml, 10 ng/ml and 100 ng/ml). KCM, keratinocyte culture medium; SA, *S. aureus*. Data are means ± SD. (*n* = 3, *p < 0.05).

**FIGURE 3:**
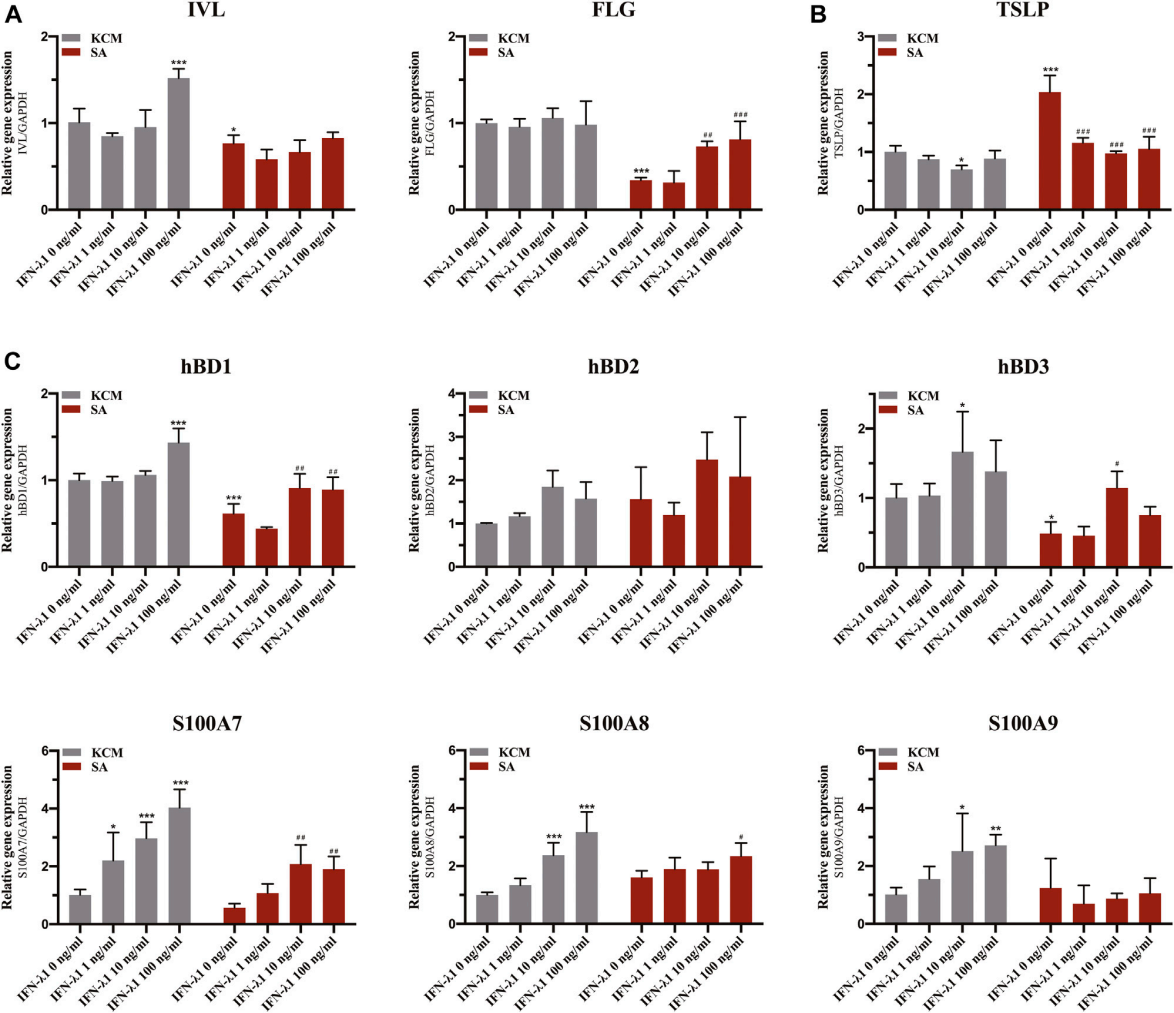
Effects of IFN-λ1 on mRNA expression of FLG, IVL, TSLP and antimicrobial peptides in *S. aureus* infected human keratinocytes. **(A)** mRNA expression of FLG and IVL; **(B)** mRNA expression of TSLP; **(C)** mRNA expression of antimicrobial peptide (hBD1, hBD2, hBD3, S100A7, S100A8, S100A9). The IFN-λ1 concentration was set as 1 ng/ml, 10 ng/ml and 100 ng/ml. KCM, keratinocyte culture medium; SA, *S. aureus*. Data are means ± SD. (*n* = 3, *p < 0.05, **p < 0.01, ***p < 0.001 vs. TCM; ^#^ *p* < 0.05, ^##^ *p* < 0.01, ^###^ *p* < 0.001 vs. SA).

FLG and IVL gene expression were evaluated in human keratinocytes. To eliminate the confounding factors irrelevant to keratinocyte proliferation, we performed cell proliferation assay. IFN-λ1 of 1, 10 and 100 ng/ml had no effect on cell proliferation (Figures 2A–C). *S. aureus* infection induced IVL and FLG mRNA significant downregulation without marked cell death (*p* < 0.05) (Figures 2D, 3A). In non-infection model, IFN-λ1 100 ng/ml increased IVL mRNA expression (Figure 3A). In infection model, keratinocytes exposed to 10 and 100 ng/ml IFN-λ1 for 24 h induced significant FLG mRNA expression (Figure 3A).

*Staphylococcus aureus* infection induced significant elevation in TSLP level (*p* < 0.05) (Figure 3B). Pre-treatment of infected keratinocytes for 24 h with IFN-λ1 significantly inhibited the TSLP level (*p* < 0.05) (Figure 3B).

### IFN-λ1 Enhances Antimicrobial Peptide Expression in Human Primary Keratinocytes Upon *Staphylococcus aureus* Infection

Keratinocytes were incubated for 24 h with IFN-λ1 prior to *S. aureus* infection. In the non-infection model, the S100A7 mRNA expression was significantly elevated with increasing IFN-λ1 concentration. S100A8 and S100A9 mRNA expression were increased in 10 ng/ml and 100 ng/ml IFN-λ1 groups. hBD1 mRNA expression was up-regulated by high concentration of IFN-λ1 (100 ng/ml). hBD3 mRNA expression was increased in 10 ng/ml IFN-λ1 group (Figure 3C). A substantial decrease in hBD1 and hBD3 mRNA expression were observed when cells were infected with *S. aureus* (*p* < 0.05) (Figure 3C). After infection, hBD1, hBD3 and S100A7 mRNA expression in IFN-λ1 10 ng/ml group were significantly increased. hBD1, S100A7, and S100A8 mRNA expression in IFN-λ1 100 ng/ml group were also significantly increased (*p* < 0.05) (Figure 3C). hBD2 remained unchanged upon *S. aureus* in each group (Figure 3C).

### IFN-λ1 Inhibits *Staphylococcus aureus* Colonization via IL-28RA-ROS-JAK-STAT1 Signaling Pathway in Human Primary Keratinocytes

**FIGURE 4:**
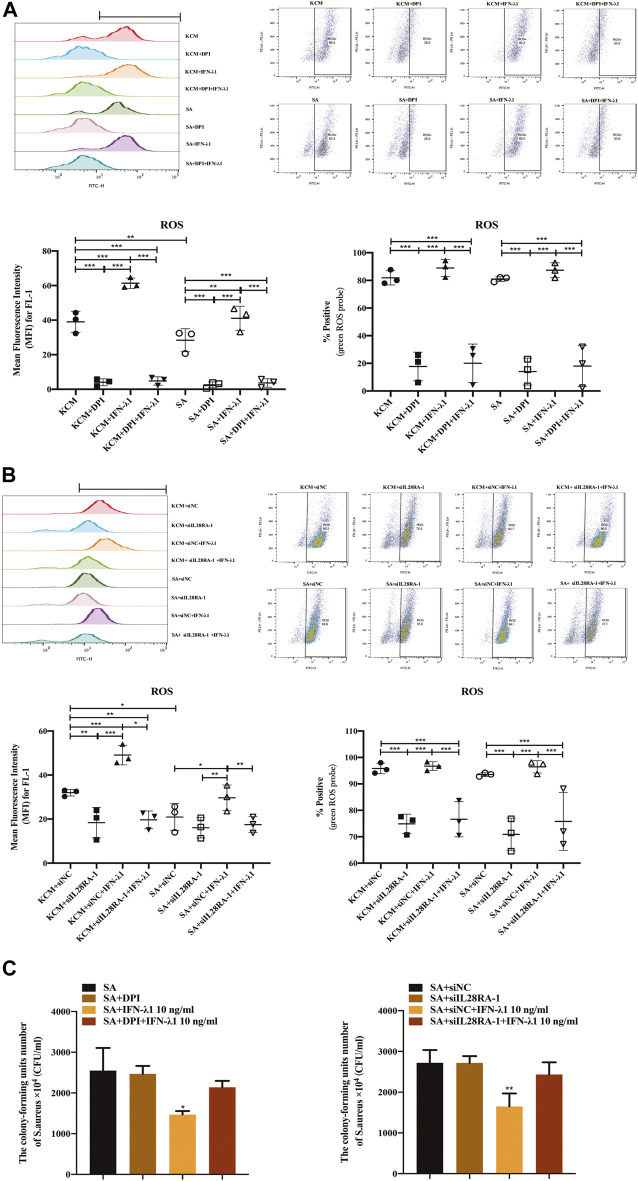
IFN-λ1 promotes ROS release and inhibits *S. aureus* colonization. **(A,B)** Effects of IFN-λ1 on ROS expression in human keratinocytes by flow cytometry. **(A)** NADPH oxidase inhibitor (DPI) blocked ROS expression significantly; **(B)** Transfection of siIL28RA-1 inhibited ROS expression significantly; **(C)** Inhibition of *S. aureus* colonization by IFN-λ1. Keratinocytes infected with *S. aureus* were lyzed with sterile water. The lysates were plated on mannitol salt agar plates for 24 h. The CFU was counted. ROS, reactive oxidase substrates; KCM, keratinocyte culture medium; SA, *S. aureus*; DPI, diphenyleneiodionium chloride; siNC, siRNA negative control; siIL28RA-1, IL-28RA1 siRNA; CFU, colony forming units; Data are means ± SD. (*n* = 3, *p < 0.05, **p < 0.01, ***p < 0.001 vs KCM or KCM + siNC; ^#^ *p* < 0.05, ^##^ *p* < 0.01, ^###^ *p* < 0.001 vs. SA or SA + siNC).

**FIGURE 5:**
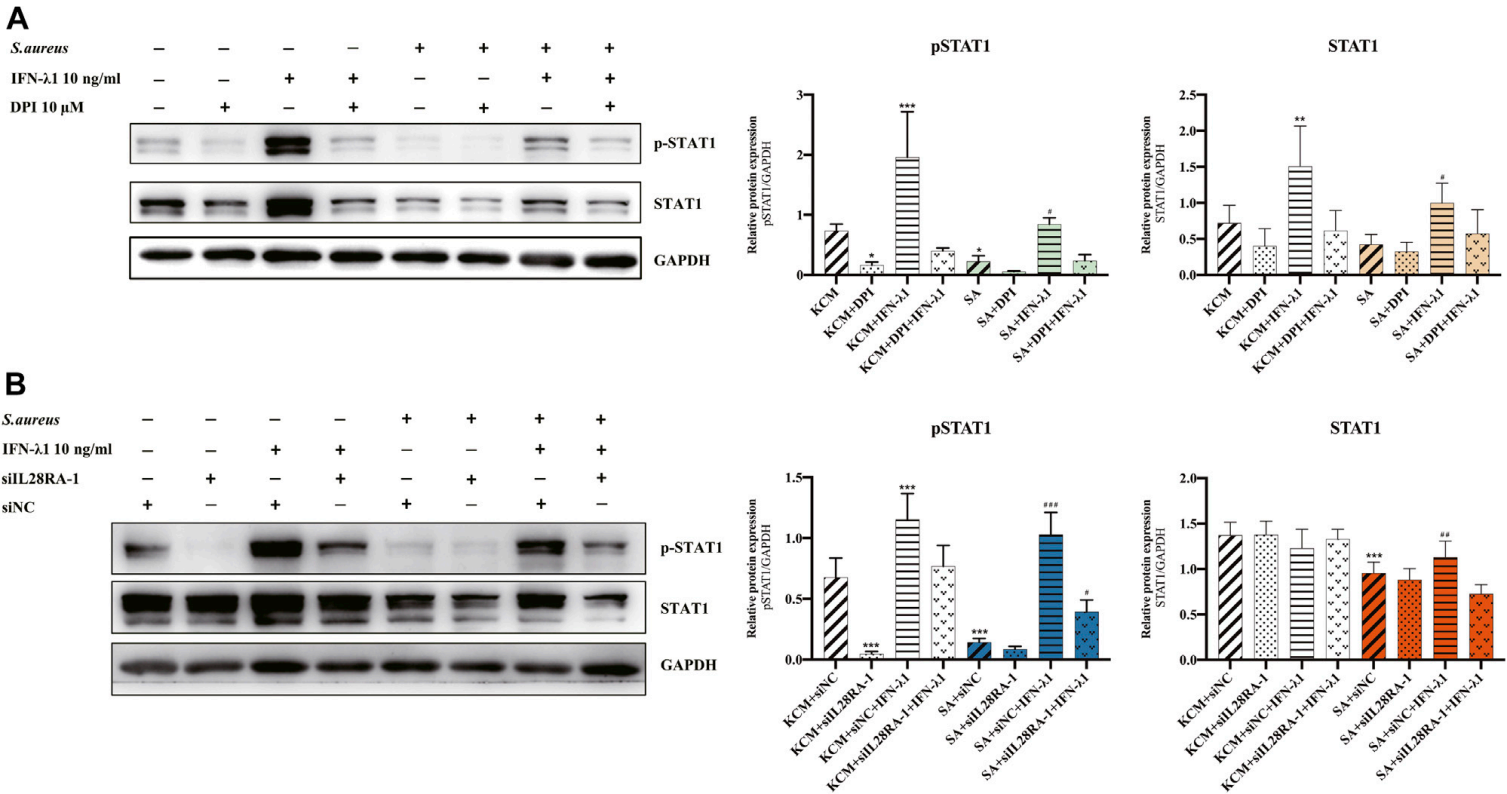
IFN-λ1 promote STAT1 phosphorylation in keratinocytes. **(A,B)** Effects of IFN-λ1 on STAT1 and pSTAT1 level. **(A)** NADPH oxidase inhibitor (DPI) inhibited pSTAT1 level significantly; **(B)** Transfection of siIL28RA-1 inhibited pSTAT1 level significantly; KCM, keratinocyte culture medium; SA, *S. aureus*; DPI, diphenyleneiodionium chloride; siNC, siRNA negative control. Data are means ± SD. (*n* = 3, *p < 0.05, **p < 0.01, ***p < 0.001 vs. KCM or KCM + siNC; ^#^ *p* < 0.05, ^##^ *p* < 0.01, ^###^ *p* < 0.001 vs. SA or SA + siNC).

To study the effects of IFN-λ1 on keratinocyte activation, we measured the ROS oxidase level which represents the microbicidal function of keratinocytes. IFN-λ1 (10 ng/ml) induced a substantial increase in IL-28RA expression infected keratinocytes (*p* < 0.05). IFN-λ1 significantly increased mean fluorescence intensity (MFI) and the percentage of positive probe for intracellular ROS levels, regardless of *S. aureus* infection (*p* < 0.05) (Figure 4A; Supplementary Figure S5A). Moreover, IFN-λ1 treatment significantly increased intracellular ROS levels and inhibited the attachment and the penetration of *S. aureus* (Figure 4C). *S. aureus* infection alone reduced MFI of ROS (*p* < 0.05) (Figure 4A). The level of pSTAT1 and STAT1 were significantly elevated by IFN-λ1 treatment regardless of *S. aureus* infection (*p* < 0.05) (Figure 5A).

NADPH oxidase inhibitor (DPI) successfully blocked IFN-λ1-induced antibacterial effects (Figure 4C). The MFI and the percentages of positive probe of ROS were down-regulated (*p* < 0.05) (Figure 4A, Supplementary Figure S5A). A four-fold decrease in pSTAT1 in keratinocytes was observed in the DPI group, compared to control (Figures 5A,B).

Experiments of transfection showed that siIL28RA-1 had the best knockdown ability and significantly inhibited IL-28RA expression and pSTAT1/STAT1 level in keratinocytes (*p* < 0.05) (Supplementary Figures S4B,C). The knockdown of IL-28RA inhibited MFI and the percentages of positive probe of ROS without affecting *S. aureus* colonization (*p* < 0.05) (Figures 4B,C; Supplementary Figure S5B). A fourteen-fold decrease in pSTAT1 in keratinocytes were observed in siIL28RA-1 transfection group, compared to control (Figure 5B).

## Discussion

It is well-established that IFN-λ represent an important frontline defense in barrier epithelia that fights against viral infections (Krug et al., 2020; Galani et al., 2021). Growing evidence has revealed new functions of IFN-λ, such as antibacterial, antifungal, and immunoregulatory functions. The effect of IFN-λ1 on anti-*S. aureus* infection in the nasal mucosa and anti-*Fetoplacental Listeriosis* in placenta was also reported (Bierne et al., 2012; Lan et al., 2019). We studied the possible anti-bacterial role of IFN-λ1 in human keratinocytes. We found that IFN-λ1 could enhance the skin barrier gene and APM gene in keratinocytes exposed to *S. aureus*, keeping in accordance with the bacterial clearance function of the epidermal barrier. IFN-λ1 can also regulate TSLP expression in keratinocytes infected by *S. aureus* to keratinocytes. Our study also demonstrated that the antimicrobial effects of IFN-λ1 in keratinocytes might be via inhibition of attachment and penetration of *S. aureus*. In addition, IFN-λ1 enhanced ROS production and STAT1 phosphorylation in keratinocytes irrespective of the presence of *S. aureus*. IL-28RA knockdown and ROS inhibition significantly inhibited the STAT1 phosphorylation. We hypothesize that IFN-λ1 might perform its anti-bacterial function by 1) restoring the epidermis barrier function and modulating keratinocyte inflammation; 2) inhibiting *S. aureus* colonization via the IL-28R-ROS-JAK-STAT1 signaling pathway.

Skin is of importance for protecting the body from external microbial invasion and allergen stimulation. Previous studies demonstrated that Th2-type cytokines can promote *S. aureus* colonization in AD skin due to impaired skin barrier function and reduced production of antimicrobial peptide (Callewaert et al., 2020; Leung et al., 2020). Other studies also suggested that impaired skin barrier contributed to childhood asthma, food allergy, and allergic rhinosinusitis, indicating the importance to restore the skin barrier function in this disease (van den Oord and Sheikh, 2009). The relationship between IFN-λs and epithelial barrier protection in various organs, e.g. respiratory tract, lungs, and gastrointestinal tract had been well-established (Lozhkov et al., 2020; Stanifer et al., 2020). The *ex vivo* infection model showed that the barrier gene FLG and IVL were generally inhibited in *S. aureus* infection model. We focused on the barrier repair function of keratinocytes in *S. aureus* infection model and displayed that IFN-λ1 could promote FLG expression in infected keratinocytes. This finding suggests that IFN-λ1 may play a role in barrier function repairment by upregulating the FLG expression, thereby protecting the epidermis from invasion and colonization of *S. aureus*.

It is known that Th2-type cytokines (IL-4, IL-5, and IL-13) and epithelial-derived innate type-2 cytokines (TSLP) increased in skin lesions of acute stage of AD (DeVore et al., 2020; Edslev et al., 2020; Langan et al., 2020). TSLP is crucial for regulating the downstream IL-4/IL-13 and Th2 differentiation. In addition, *S. aureus* inhibited STAT1 phosphorylation and contributed to downregulation of CXCL9 and CXCL10 in monocytes (Li et al., 2017). It has been reported that IFN-λs can inhibit Th2 cytokine production in human peripheral blood mononuclear cells (PBMCs) (Jordan et al., 2007; Srinivas et al., 2008) and stimulate Th1 chemokines (CXCL9, CXCL10 and CXCL11) production in HaCaT cells (Syedbasha and Egli, 2017; Goel et al., 2020). In addition, IFN-λ2/3 can inhibit TSLP secretion in bronchoalveolar lavage fluid of asthmatic mice (Won et al., 2019). Our findings indicate that IFN-λ1 can also inhibt the secretion of TSLP, which might contribute to the alleviation of skin inflammation.

Skin lesions of AD are routinely associated with microbial colonization (*S. aureus* and HSV). The diminished expression of IFN-λ1 and AMP was found in skin lesions of AD (Ong et al., 2002; de Jongh et al., 2005; Wolk et al., 2013). Bin et al. (Bin et al., 2014) reported that IFN-λ1 was downregulated in HSV-1 infected PBMCs and in culture supernatants of PBMC of eczema herpeticum. This phenomenon was absent in AD patients without eczema herpeticum and in normal controls. We found that IFN-λ1 can affect the AMP expression in keratinocytes infected with *S. aureus*. We also found the anti-bacterial function of IFN-λ1 was via IL-28R-ROS-JAK-STAT signaling pathway. This was supported by increasing ROS levels and STAT1 phosphorylation in infected keratinocytes by IFN-λ1 treatment.

In conclusion, we found that IFN-λ1 facilitates the clearance of *S. aureus* in human epidermis keratinocytes through the IL28RA-ROS-JAK-STAT1 pathway. It also impacted the skin barrier gene and AMP secretion, and inhibited TSLP expression. This might contribute to antibacterial function of epidermis keratinocytes and reduction of skin inflammation.

## Data Availability

The original contributions presented in the study are included in the article/Supplementary Material, further inquiries can be directed to the corresponding author.
