# Supplementary material for: Interferon-Lambda 1 Inhibits Staphylococcus aureus Colonization in Human Primary Keratinocytes
Source: Front Pharmacol. 2021 Mar 22;12:652302. doi: 10.3389/fphar.2021.652302 (PMC8019897; doi:10.3389/fphar.2021.652302)
Supplement: Supplementary file 1 [file datasheet1.docx]

# Interferon-Lambda 1 Inhibits *Staphylococcus aureus* Colonization in Human Primary Keratinocytes

Xia Wu et al.

**Supplementary Tables**

**Table S1. The primers sequence used for *S. aureus* multiplex PCR.**

| Primer name | Sequence (5’ – 3’) | bp |
| --- | --- | --- |
| 16s rRNA-Forward | CGTGCCTAATACATGCAAGTCG | 1322 |
| 16s rRNA-Reverse | TACGATTACTAGCGATTCCAGC |  |
| femA-Forward | AACTGTTGGCCACTATGAGT | 306 |
| femA-Reverse | CCAGCATTACCTGTAATCTCG |  |
| mecA-Forward | TAGTTGTAGTTGTCGGGT | 167 |
| mecA-Reverse | TATCGGACGTTCAGTCAT |  |

**Table S2. The primers sequence used for qRT-PCR.**

| Primer name | Sequence (5’ – 3’) | bp |
| --- | --- | --- |
| IFN-λ1-Forward | TGGGAACCTGTGTCTGAGAACG | 70 |
| IFN-λ1-Reverse | AGGGCTCAGCGCATAAATAAGGTG |  |
| IL-28RA-Forward | CCTCCCCAGAATGTGACGC | 152 |
| IL-28RA-Reverse | CCCGCACACTCTTCCACTT |  |
| IL-10RB-Forward | ATGAGCATTCAGACTGGGTAAAC | 123 |
| IL-10RB-Reverse | TTTTAGGGGCTAAGAAACGCAT |  |
| IVL-Forward | ACAAGGGAAGAGAGAGCCACTG | 88 |
| IVL-Reverse | TGTAGAGGGACAGAGTCAAGTTC |  |
| FLG-Forward | TGAAGCCTATGACACCACTGA | 160 |
| FLG-Reverse | TCCCCTACGCTTTCTTGTCCT |  |
| TSLP-Forward | CCCAGGCTATTCGGAAACTCAG | 117 |
| TSLP-Reverse | CGCCACAATCCTTGTAATTGTG |  |
| hBD1-Forward | ATGAGAACTTCCTACCTTCTGCT | 183 |
| hBD1-Reverse | TCTGTAACAGGTGCCTTGAATTT |  |
| hBD2-Forward | TGTGGTCTCCCTGGAACAAAAT | 105 |
| hBD2-Reverse | GTCGCACGTCTCTGATGAGG |  |
| hBD3-Forward | TTATTGCAGAGTCAGAGGCGG | 105 |
| hBD3-Reverse | TTTCTTCGGCAGCATTTTCGG |  |
| S100A7-Forward | ACGTGATGACAAGATTGACAAGC | 102 |
| S100A7-Reverse | GCGAGGTAATTTGTGCCCTTT |  |
| S100A8-Forward | ATGCCGTCTACAGGGATGAC | 54 |
| S100A8-Reverse | ACTGAGGACACTCGGTCTCTA |  |
| S100A9-Forward | GGTCATAGAACACATCATGGAGG | 155 |
| S100A9-Reverse | GGCCTGGCTTATGGTGGTG |  |

**Supplementary Figure legends:**


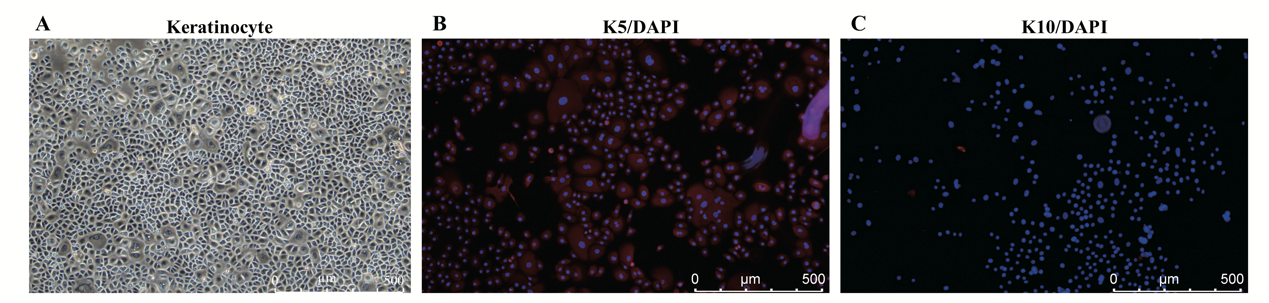


**Figure S1**. (A) The representative images of the primary epidermal keratinocyte; (B) the immunofluorescence staining of antibodies to keratin 5 (K5, red) and DAPI (blue); (C) the immunofluorescence staining of antibodies to keratin 10 (K10, red) and DAPI (blue).


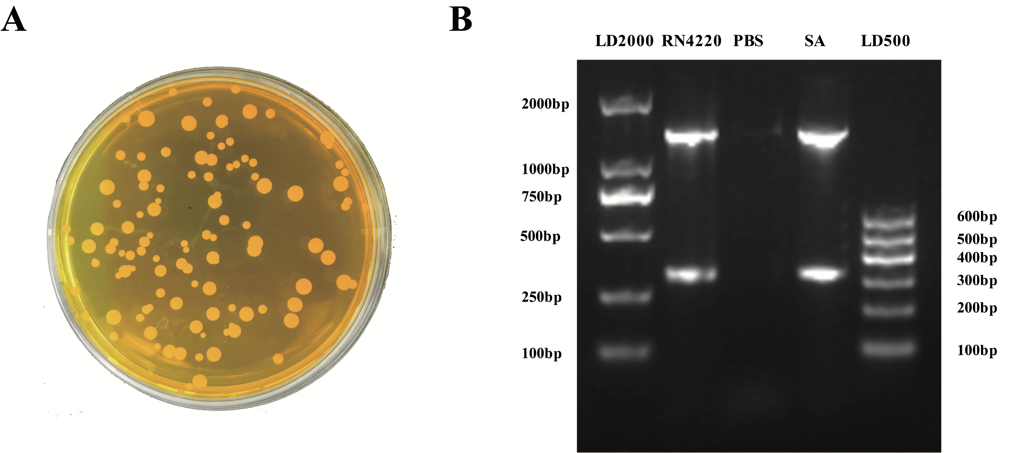


**Figure S2**. (A) *S. aureus* were plated onto the mannitol salt agar (MSA) plate; (B) Amplification results of 16S rRNA gene, femA gene and mecA gene from RN4220 (positive control), PBS (negative control) and SA; SA, *S. aureus* (marker:100-2000bp and 100–600 bp).


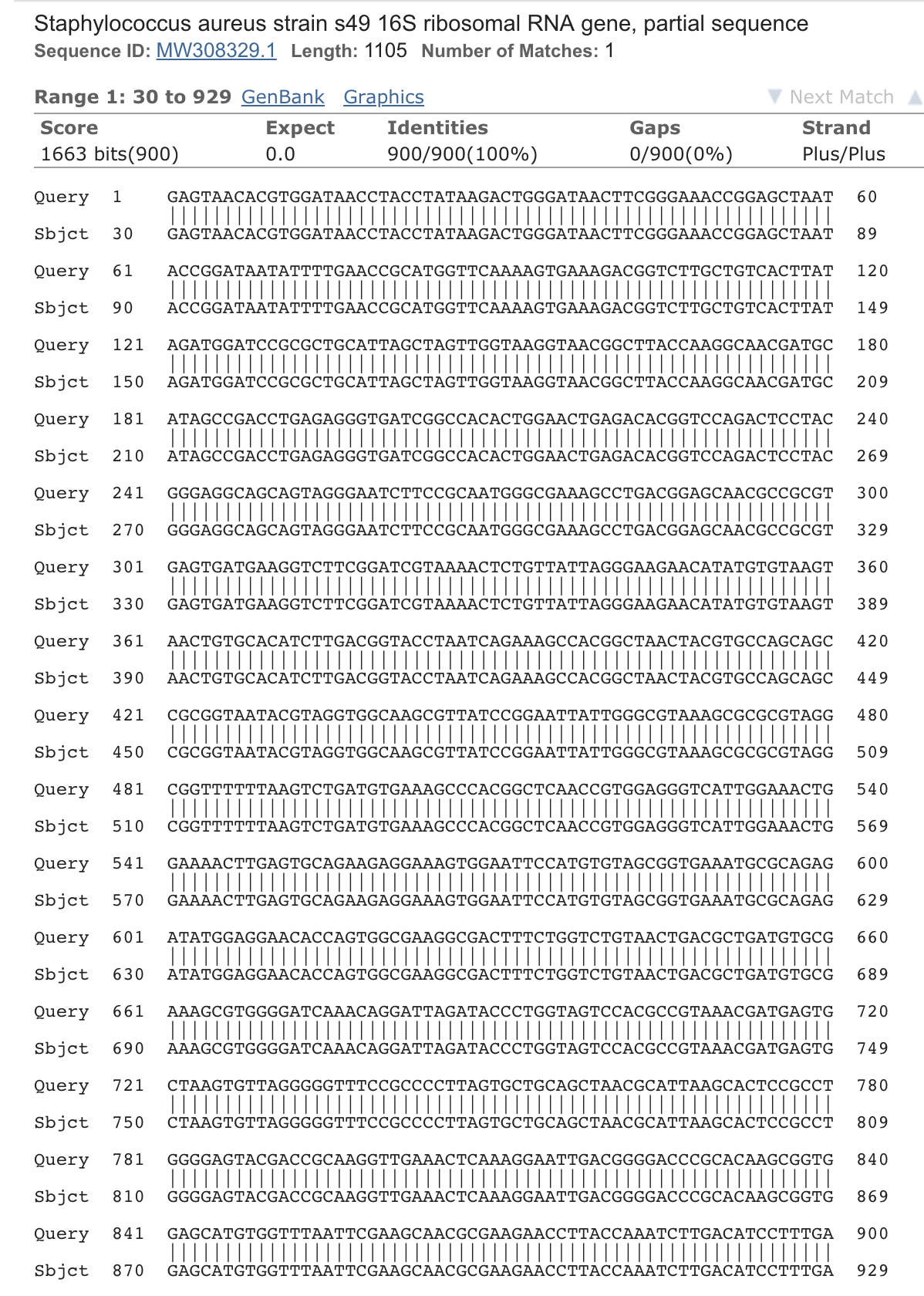


**Figure S3**. Comparative analysis of the 16S rRNA sequence isolated in this study and the existing *S. aureus* strains sequence


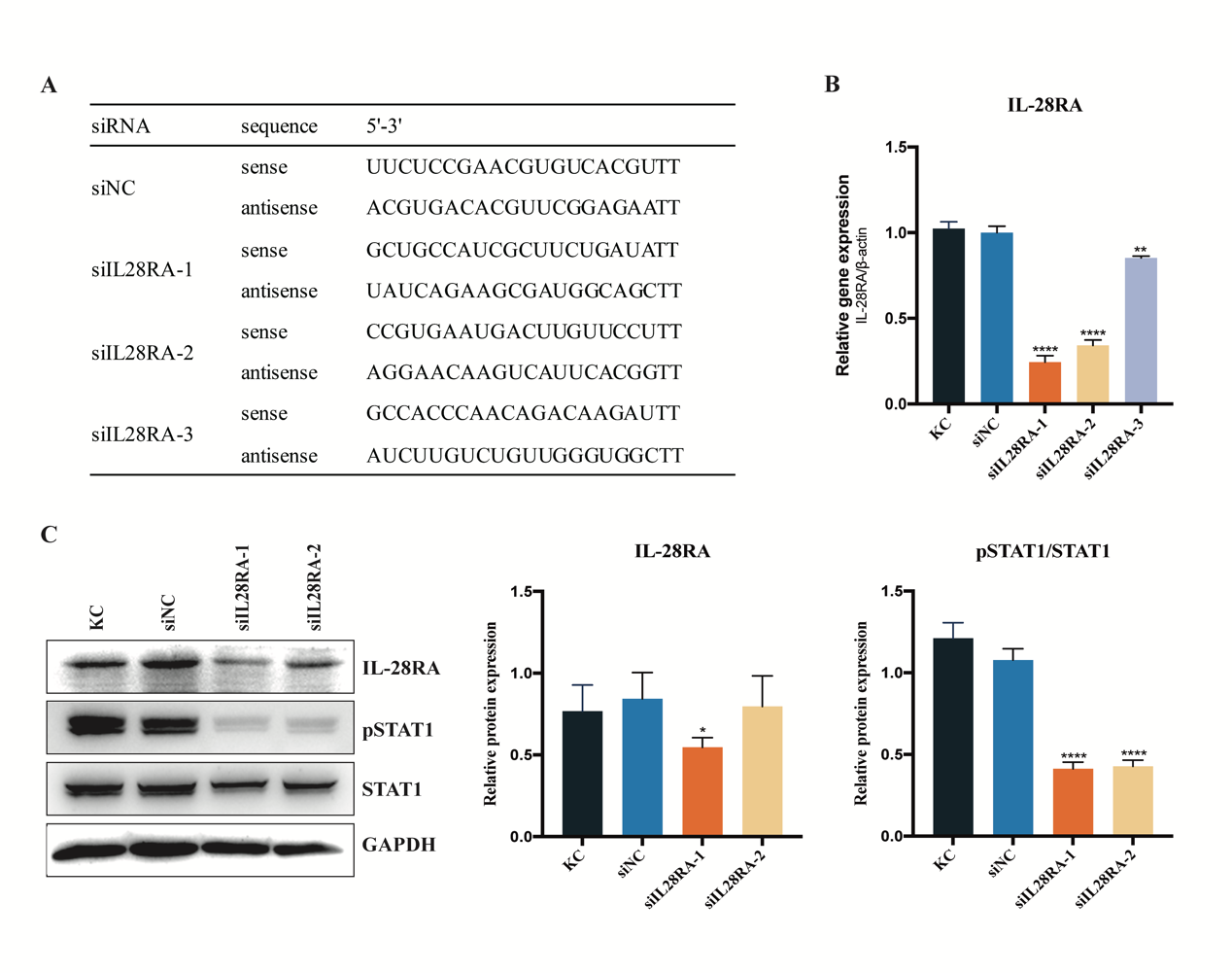


**Figure S4. IL-28RA knock-down induces pSTAT1/STAT1 inhibition.** (A) The siRNA sequences of IL-28RA used in this study. (B) The relative IL-28RA mRNA expression after siIL-28RA-1, siIL-28RA-2 and siIL-28RA-3 transfection compared to siNC and KC. (C) The relative protein expression of IL-28RA, p-STAT1 and STAT1 after siIL-28RA-1 and siIL-28RA-2 transfection compared to siNC and KC. KC, keratinocyte. Data are means ± SD. (n=3, **p* < 0.05, ***p* < 0.01, ****p* < 0.001)


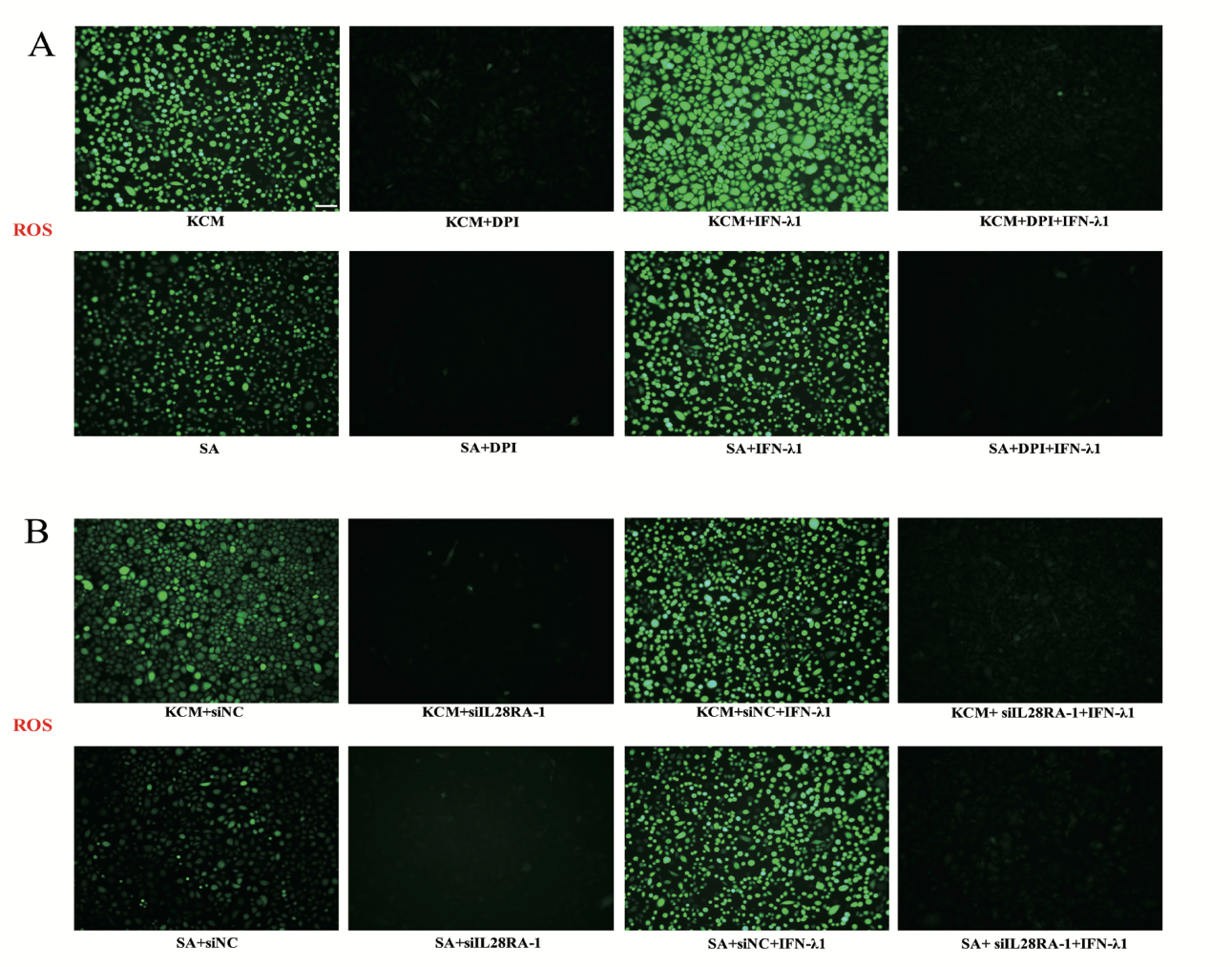


**Figure S5. IFN-λ1 promotes ROS release.** (A, B) Effects of IFN-λ1 on ROS expression in human keratinocytes by fluorescence microscopy. (A) NADPH oxidase inhibitor (DPI) blocked ROS expression; (B) Transfection of siIL28RA-1 inhibited ROS expression significantly. ROS, reactive oxidase substrates; KCM, keratinocyte culture medium; SA, *S. aureus*; DPI, diphenyleneiodionium chloride; siNC, siRNA negative control; siIL28RA-1, IL-28RA1 siRNA; CFU, colony forming units; Data are means ± SD. (n=3, **p* < 0.05, ***p* < 0.01, ****p* < 0.001 vs KCM or KCM+siNC; **^#^***p* < 0.05, **^##^***p* < 0.01, **^###^***p* < 0.001 vs. SA or SA+siNC)
